# Psychiatric comorbidity in individuals with bullous pemphigoid and all bullous disorders in the Danish national registers

**DOI:** 10.1186/s12888-020-02810-x

**Published:** 2020-08-20

**Authors:** Marianna Rania, Liselotte Vogdrup Petersen, Michael Erikson Benros, Zhi Liu, Luis Diaz, Cynthia M. Bulik

**Affiliations:** 1 Department of Health Sciences, University Magna Graecia of Catanzaro, Catanzaro, Italy; 2 Center for Clinical Research and Treatment of Eating Disorders, Mater Domini University Hospital, Catanzaro, Italy; 3 Department of Medical Epidemiology and Biostatistics, Karolinska Institutet, Stockholm, Sweden; 4 National Centre for Register-based Research, Aarhus BSS, Aarhus University, Aarhus, Denmark; 5 Centre for Integrated Register-based Research (CIRRAU), Aarhus University, Aarhus, Denmark; 6 Copenhagen Research Centre for Mental Health, Mental Health Centre Copenhagen, Copenhagen University Hospital, Copenhagen, Denmark; 7 Departments of Dermatology, Microbiology and Immunology, University of North Carolina at Chapel Hill, Chapel Hill, NC USA; 8 Department of Psychiatry, University of North Carolina at Chapel Hill, Chapel Hill, NC USA; 9 Department of Nutrition, University of North Carolina at Chapel Hill, Chapel Hill, NC USA

**Keywords:** Bullous pemphigoid, Bullous disorders, Mental health, Psychiatric disorders, Comorbidity

## Abstract

**Background:**

Bullous pemphigoid (BP) is an autoimmune blistering skin disease that takes a profound physical and mental toll on those affected. The aim of the study was to investigate the bidirectional association between BP and all bullous disorders (ABD) with a broad array of psychiatric disorders, exploring the influence of prescribed medications.

**Methods:**

This nationwide, register-based cohort study encompassed 6,470,450 individuals born in Denmark and alive from 1994 to 2016. The hazard ratios (HRs) of a subsequent psychiatric disorder in patients with BP/ABD and the reverse exposure and outcome were evaluated.

**Results:**

Several psychiatric disorders were associated with increased risk of subsequent BP (4.18-fold for intellectual disorders, 2.32-fold for substance use disorders, 2.01-fold for schizophrenia and personality disorders, 1.92–1.85-1.49-fold increased risk for organic disorders, neurotic and mood disorders), independent of psychiatric medications. The association between BP and subsequent psychiatric disorders was not significant after adjusting for BP medications, except for organic disorders (HR 1.27, CI 1.04–1.54). Similar results emerged with ABD.

**Conclusion:**

Psychiatric disorders increase the risk of a subsequent diagnosis of BP/ABD independent of medications, whereas medications used for the treatment of BP/ABD appear to account for the subsequent onset of psychiatric disorders. Clinically, an integrated approach attending to both dermatological and psychiatric symptoms is recommended, and dermatologists should remain vigilant for early symptoms of psychiatric disorders to decrease mental health comorbidity.

## Background

Bullous pemphigoid (BP) is categorized among a heterogeneous panel of bullous disorders (ABD), and is characterized clinically by itchy and tense serous or hemorrhagic sub-epidermal blisters that can be localized or widespread on the trunk and extremities and typically affects older individuals [1, 2].

The incidence of BP in Europe is estimated to be 2.5–42.8 cases/million/year [3–9], with incidence increasing over the past eight decades [10] and a nearly 300-fold increase in those over age 90 compared to those younger than 60 [4]. This rise may reflect increasing average life expectancy, increasing use of polypharmacy, and improved detection and diagnosis [5]. BP is associated with considerable functional impairment and distress [11, 12] and the one-year mortality rate of BP in Europe is ~ 26.7%, although other factors should be considered (high somatic comorbidity and polypharmacy) [13].

BP is an organ-specific autoimmune disease mediated by IgG autoantibodies that target two epidermal hemidesmosomal antigens crucial for adhesion between epidermis and dermis, BP230 (or BP Ag1/dystonin-e) [14],and BP180 (or BP Ag2/collagen XVII) [15, 16]. These autoantibodies induce sub-epidermal blisters in the skin by triggering an inflammatory response mediated by complement activation, mast cells, eosinophils, and neutrophils [2, 17].

BP antigens have been identified in brain and neuronal tissue [18, 19] and high levels of autoantibodies against BP antigens have been found in individuals affected by other neurological disorders [20–22]. Significant associations have been reported between BP and neurological diseases [23, 24] and even if mechanisms underlying this association are unclear, brain-reactive antibodies have been detected in the sera of individuals with BP, which might account for the association [22, 25].

To date, only few studies have explored psychiatric comorbidity in individuals with BP, and significantly elevated odds ratios (OR) have been reported for schizophrenia (OR range 1.25–2.7) [26–28], major depressive disorder, and bipolar disorder (OR range 1.19–5.25) [28, 29], although these observations have not been universally replicated [30].

Försti et al. [27] conducted a large nationwide retrospective study on the association between BP and neurological and psychiatric disorders. BP patients were at increased risk for schizotypal and delusional disorders, schizophrenia, bipolar affective disorder, major depressive disorder, neurotic, stress related and somatoform disorders, and personality disorders (ORs 1.27–2.7), with higher risk of BP after receiving a psychiatric diagnosis than vice-versa. However, this study was restricted to psychiatric diagnoses recorded in specialized settings and included patients with basal cell carcinoma as control group.

Importantly, medications used to treat BP, primarily corticosteroids, have been associated with the onset of psychiatric disorders [31, 32] and some antipsychotic, antidepressant, and anxiolytic medications may increase risk for BP/ABD [29, 33–36]. Many questions remain regarding the association between BP/ABD and psychiatric disorders that can best be addressed by population health registers with universal diagnostic detection and prescription records.

In order to fully address the nature of the relation between BP/ABD and psychiatric disorders, we conducted a comprehensive register-based investigation of lifetime co-occurrence of BP/ABD and a broad array of psychiatric illnesses using the Danish nationwide registers to explore the nature and direction of the association, and the extent to which medications prescribed to treat each class of illness influences any observed relation.

## Methods

### Study design

We examined the association between BP/ABD and psychiatric disorders. To address temporality, we first estimated the risk for individuals with each psychiatric disorder to receive a later diagnosis of BP/ABD. We then reversed the question and determined the risk of developing any psychiatric disorder after a diagnosis of BP/ABD. Individuals with a diagnosis of BP, ABD, or psychiatric disorder, either existing at the beginning or incident during the observational period, were considered exposed, compared to the general population. As outcomes, only incident cases of BP, ABD, or psychiatric disorders during the follow-up were considered as subsequent disorders, accordingly, each analysis had different number of individuals at risk at baseline. Observations commenced on January 1, 1994 and stopped with the outcome, emigration, death or end of study period, on December 31st 2016, whichever came first.

### Study population and assessment

Since 1968, the Danish Civil Registration System [37] includes information on each resident in Denmark regarding place and date of birth, sex, and vital status and assigns all Danish residents a unique personal identification number enabling linkage to the national registers of somatic and psychiatric disorders.

Cases of BP/ABD were identified via the Danish National Patient Register [38], which includes all diagnoses entered in Danish general hospitals since 1977, and all outpatient contacts since January 1, 1995. According to the International Statistical Classification of Diseases and Related Health Problems, 10th Revision (ICD-10), L12.0, L12.8–12.9 diagnostic codes were considered BP cases, and L10-L14 were considered ABD cases.

Psychiatric diagnoses were obtained from the Danish Psychiatric Central Research Register [39], which includes all hospitalizations in psychiatric hospitals since 1969 and outpatient treatment and emergency room contacts since 1995. We considered all diagnoses included in the F subchapter of ICD-10 (Mental and behavioral disorders; F00–98). We use abbreviations to refer to psychiatric disorders, in accordance with Plana-Ripoll et al. [40]

Prescription medication data were obtained through the National Prescription Registry [41], which contains information on all redeemed prescriptions since January 1, 1995. Medications typically prescribed for the treatment of BP and ABD (H02, systemic corticosteroids; J01A, tetracyclines; L04AX01, azathioprine; L04AX03, methotrexate; L01AA01, cyclophosphamide; L04AD01, cyclosporine; L04AA06, mycophenolate mofetil), psychiatric disorders (N05A-B-C, psycholeptics; N06A, antidepressants; N03A, mood stabilizers/antiepileptics), and medications known to be associated with drug-induced BP (specifically diuretics; C03CA01, furosemide; C03CB01, furosemide and potassium; C03EB01, furosemide and potassium-sparing agents; C03DA01, spironolactone) were obtained using Anatomical Therapeutic Chemical (ATC) codes [42]. All personal data from the registers is anonymized when used for research purposes, and the study was approved by the Danish Data Protection Agency and Statistics Denmark, necessitating no informed consent.

### Statistical analysis

We estimated the hazard of developing a psychiatric disorder in patients with BP/ABD compared to the general population, by including BP/ABD as time dependent variable, and then reversed exposure and outcome, by including each psychiatric disorder as a time dependent variable.

For both sets of analyses, estimates were obtained via Cox regression with the underlying hazard as function age allowed to differ by sex. We present results as hazard ratios (HR) with 95%-confidence intervals (95%CI). Analyses were adjusted for birth decade and calendar year (1994–1999, 2000–2004, 2005–2009, and 2010–2016) as time dependent variables. Adjustment for medication was handled as a time dependent variable on the day a second prescription for the drug of interest was redeemed.

Analyses were conducted between January and October 2019 and were performed with Stata version 15 [43].

## Results

A total of 6,470,450 individuals were followed from 1994 to 2016. Overall, 1,249,903 individuals died, 117,690 emigrated from Denmark, and 1851 disappeared during the observation period.

Table 1 shows characteristics of the study population.

**Table 1:** Characteristics of the sample

**Bullous pemphigoid (BP)**				
	BP and no psychiatric diagnosis	Psychiatric diagnosis after BP	Psychiatric diagnosis and no BP	BP diagnosis after psychiatric disorder
N^a^	2370	122	506,354	400
Onset of BP mean (SD)	77.0 (13.4)	76.9 (12.4)	–	75.6 (12.5)
Birth year mean (SD)	1929 (15)	1927 (13)	1965 (27)	1931 (14)
Percentage women (%)	53.9	56.6	54.2	64.5
Onset psychiatric disorder mean (SD)	–	80.3 (12.0)	40.1 (23.7)	60.1 (18.9)
**All bullous disorders (ABD)**				
	ABD and no psychiatric diagnosis	ABD before psychiatric diagnosis	Psychiatric diagnosis and no ABD	Psychiatric diagnosis before ABD
N^a^	4650	236	506,028	694
Onset of BD mean (SD)	66.2 (22.7)	62.9 (27.6)	–	69.5 (16.9)
Birth year mean (SD)	1941 (24)	1940 (27)	1965 (27)	1938 (19)
Percentage women (%)	52.7	57.2	54.2	63.1
Onset psychiatric disorders mean (SD)	–	67.6 (25.3)	40.6 (25.8)	54.0 (20.9)

^a^Note survival bias. In the population with neither BP/ABD nor any psychiatric diagnoses including more than 5,767,468/5,764,277 million people, birth year (SD): 1967 (29) and 49.6% women

### The association between BP and psychiatric disorders

The most common psychiatric comorbidities were organic disorders, mood disorders, and neurotic disorders. Among individuals with BP (*N* = 2892), the mean latency until later psychiatric disorders was 4.8 years, and the risk was significantly elevated for the overall category of psychiatric disorders (HR 1.37, CI 1.15–1.63), and specifically for organic disorder (HR 1.35, CI 1.10–1.64), and mood disorder (HR 1.55, CI 1.12–2.15), compared to individuals without BP. When adjusting for BP medications and diuretics, only the association between BP and organic disorders (HR 1.27, 1.04–1.54) remained significant.

**Fig. 1:**
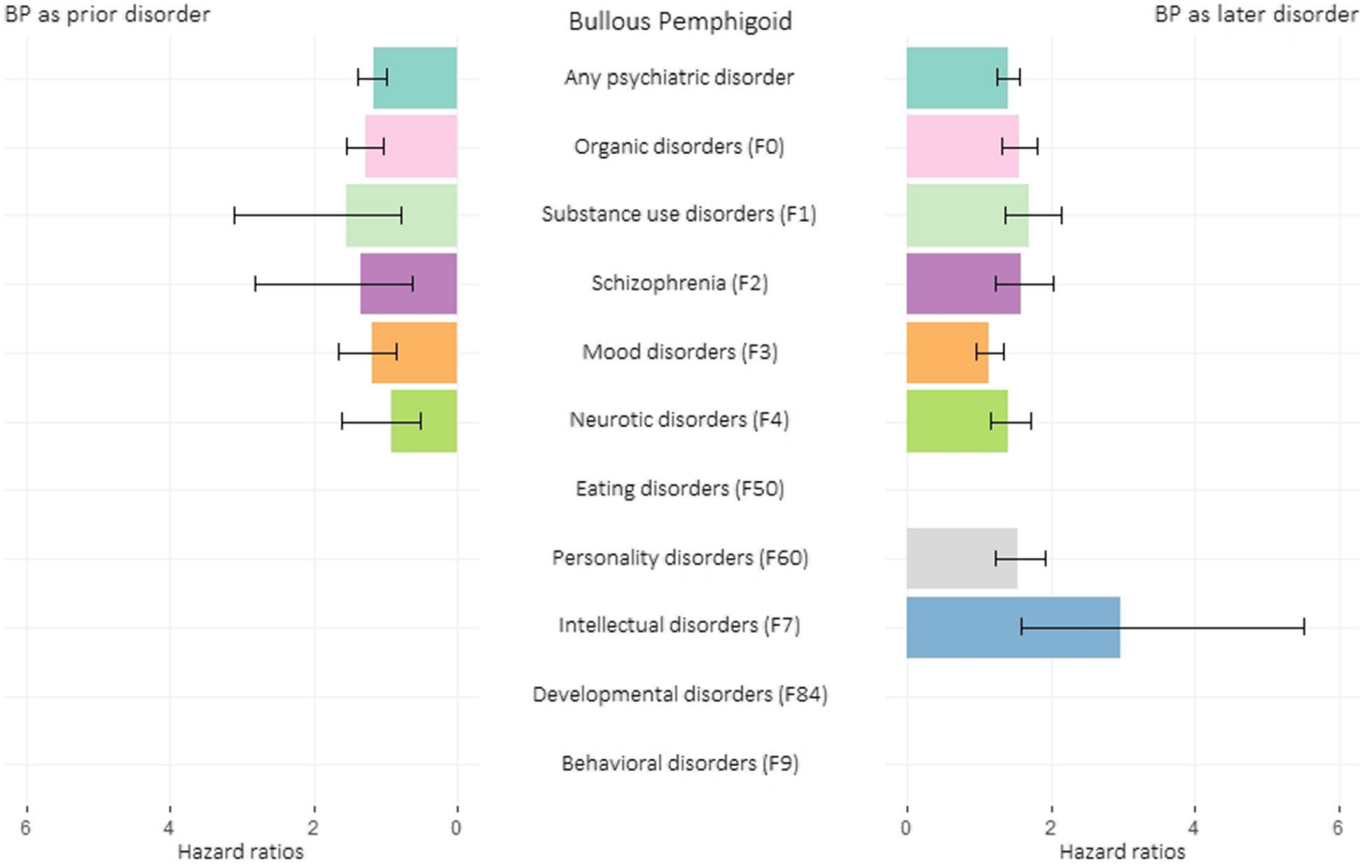
Association between bullous pemphigoid (BP) and psychiatric disorders. Bar plot showing the hazard ratios (HRs) and 95% CIs of the association between psychiatric disorders and BP, depending on whether BP is prior/later disorder. On the left side, the bars represent the HRs of receiving a diagnosis of psychiatric disorder after a previous diagnosis of BP; on the right side, the bars represent the HRs of receiving a diagnosis of BP after a previous diagnosis of psychiatric disorder. Estimates were obtained via Cox regression with age as the underlying sex-specific timescale and adjusted for medications

Among individuals with psychiatric disorders, the risk of subsequent BP was increased by 79% (HR 1.79, CI 1.61–1.99; mean latency of 14.6 years), and significantly higher for individuals with prior intellectual disorders (4.18-fold increased risk), substance use disorders (2.32-fold increased risk), schizophrenia (2.01-fold increased risk), personality disorders (2.01-fold increased risk), organic (1.92-fold increased risk), neurotic (1.85-fold increased risk), and mood disorders (1.49-fold increased risk), compared with controls. The associations remained significant after adjusting for psychiatric medications and diuretics, with the exception of the association with mood disorders (Table 2, Fig. 1).

**Table 2:** Results for bullous pemphigoid (BP) and psychiatric disorders

**BP and subsequent psychiatric disorders**^a^					
	Psychiatric diagnosis (N)	HR of psychiatric disorder	CI	HR of psychiatric disorder ^c^	CI
Any psychiatric disorder	122	1.37	1.15–1.63	1.17	0.98–1.40
Organic disorders (F0)	99	1.35	1.10–1.64	1.27	1.04–1.54
Substance use disorders (F1)	8	1.91	0.96–3.82	1.55	0.78–3.11
Schizophrenia (F2)	7	1.40	0.67–2.94	1.34	0.64–2.82
Mood disorders (F3)	36	1.55	1.12–2.15	1.20	0.86–1.66
Neurotic disorders (F4)	12	1.20	0.68–2.11	0.92	0.52–1.61
Eating disorders (F50)	< 5	–	–	–	–
Personality disorders (F60)	< 5	–	–	–	–
Intellectual disorders (F7)	< 5	–	–	–	–
Developmental disorders (F84)	< 5	–	–	–	–
Behavioral disorders (F9)	< 5	–	–	–	–
**Psychiatric disorders and subsequent BP**^b^					
	BP diagnosis (N)	HR of BP	CI	HR of BP ^d^	CI
Any psychiatric disorder	400	1.79	1.61–1.99	1.40	1.25–1.56
Organic disorders (F0)	167	1.92	1.64–2.25	1.55	1.32–1.81
Substance use disorders (F1)	79	2.32	1.86–2.91	1.70	1.35–2.13
Schizophrenia (F2)	61	2.01	1.56–2.59	1.57	1.21–2.02
Mood disorders (F3)	146	1.49	1.26–1.76	1.13	0.95–1.34
Neurotic disorders (F4)	106	1.85	1.52–2.25	1.41	1.16–1.71
Eating disorders (F50)	< 5	–	–	–	–
Personality disorders (F60)	79	2.01	1.61–2.52	1.53	1.22–1.92
Intellectual disorders (F7)	10	4.18	2.25–7.78	2.96	1.58–5.51
Developmental disorders (F84)	< 5	–	–	–	–
Behavioral disorders (F9)	< 5	–	–	–	–

^a^Total population: 6,276,714 of whom 506,876 develop a psychiatric disorder during 109 million person-years

^b^Total population: 6,448,090 of whom 2892 develop BP, during 115 million person-years

^c^Adjusted for BP medications and diuretics

^d^Adjusted for psychiatric medications and diuretics

### The association between ABD and psychiatric disorders

Individuals diagnosed with ABD (*N* = 5580) had elevated risk of developing any psychiatric disorder (HR 1.30, CI 1.15–1.48), organic disorder (HR 1.24, CI 1.05–1.46), mood (HR 1.49, CI 1.19–1.85), and neurotic disorder (HR 1.49, CI 1.15–1.94), with a mean latency of 10.1 years.

**Fig. 2:**
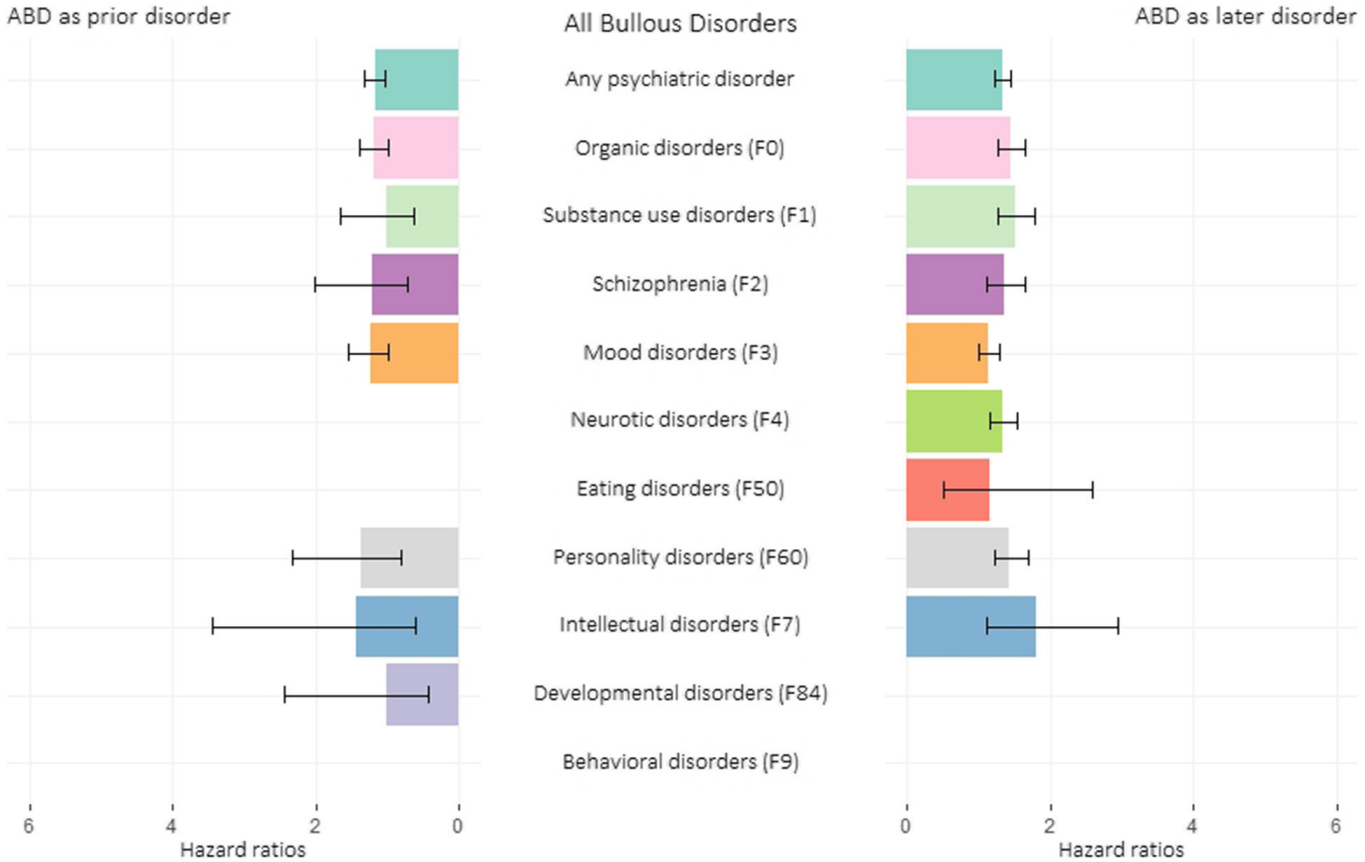
Association between all bullous disorders (ABD) and psychiatric disorders. Bar plot showing the hazard ratios (HRs) and 95% CIs of the association between psychiatric disorders and ABD, depending on whether ABD is prior/later disorder. On the left side, the bars represent the HRs of receiving a diagnosis of psychiatric disorder after a previous diagnosis of ABD; on the right side, the bars represent the HRs of receiving a diagnosis of ABD after a previous diagnosis of psychiatric disorder. Estimates were obtained via Cox regression with age as the underlying sex-specific timescale and adjusted for medications

When controlling for BP/ABD medications and diuretics, the association became non-significant, except for any psychiatric disorder. Among individuals with psychiatric disorders, the risk for subsequent ABD (mean latency of 15.4 years) was significantly higher for almost all psychiatric disorders in analysis, and independent of psychiatric medications and diuretics, with the exception of the association with mood disorders (Table 3, Fig. 2).

**Table 3:** Results for all bullous disorders (ABD) and psychiatric disorders

**ABD and subsequent psychiatric disorders**^a^					
	Psychiatric diagnosis (N)	HR of psychiatric diagnosis	CI	HR of psychiatric diagnosis ^c^	CI
Any psychiatric disorder	236	1.30	1.15–1.48	1.17	1.03–1.33
Organic disorders (F0)	143	1.24	1.05–1.46	1.18	1.00–1.39
Substance use disorders (F1)	17	1.16	0.72–1.87	1.02	0.64–1.65
Schizophrenia (F2)	15	1.24	0.75–2.06	1.21	0.73–2.01
Mood disorders (F3)	79	1.49	1.19–1.85	1.24	1.00–1.55
Neurotic disorders (F4)	56	1.49	1.15–1.94	1.29	0.99–1.67
Eating disorders (F50)	< 5	–	–	–	–
Personality disorders (F60)	14	1.54	0.91–2.60	1.38	0.81–2.32
Intellectual disorders (F7)	5	1.47	0.61–3.54	1.43	0.60–3.45
Developmental disorders (F84)	5	1.01	0.42–2.43	1.01	0.42–2.45
Behavioral disorders (F9)	< 5	–	–	–	–
**Psychiatric disorders and subsequent BP**^b^					
	ABD diagnosis (N)	HR of ABD	CI	HR of ABD^d^	CI
Any psychiatric disorder	694	1.72	1.58–1.86	1.33	1.23–1.45
Organic disorders (F0)	226	1.80	1.57–2.06	1.44	1.26–1.65
Substance use disorders (F1)	154	2.07	1.76–2.43	1.50	1.27–1.77
Schizophrenia (F2)	104	1.77	1.45–2.15	1.35	1.11–1.64
Mood disorders (F3)	251	1.49	1.31–1.69	1.12	0.99–1.28
Neurotic disorders (F4)	216	1.77	1.54–2.03	1.34	1.16–1.54
Eating disorders (F50)	6	1.49	0.66–3.33	1.15	0.51–2.58
Personality disorders (F60)	156	1.90	1.62–2.23	1.43	1.22–1.68
Intellectual disorders (F7)	16	2.52	1.54–4.12	1.80	1.10–2.95
Developmental disorders (F84)	–	–	–	–	–
Behavioral disorders (F9)	–	–	–	–	–

^a^Total population: 6,275,885 of whom 506,958 develop a psychiatric disorder during 110 million person-years

^b^Total population: 6,446,852 of whom 5580 develop ABD, during 116 million person-years

^c^Adjusted for ABD medications and diuretics

^d^Adjusted for psychiatric medications and diuretics

## Discussion

In this large comprehensive national register study, several psychiatric disorders were significantly associated with BP in specific, as well as with the broad category of ABD. Although the temporal patterns indicate bidirectionality, the risk of a psychiatric disorder preceding BP or ABD was greatest.

Our results extend the study by Försti et al. who reported an association between BP and schizophrenia, personality disorders, neurotic, and organic disorders [27]. In our study, mood disorders carried increased risk for BP (HR 1.49, CI 1.26–1.76), confirming all previous studies [11, 27, 28] except Teixeira and colleagues [30], whose small sample size and retrospective evaluation of depression may have precluded detection of significant associations.

We present a novel finding of prior intellectual disorders and substance use disorders exhibiting the highest risk of BP of all psychiatric disorders (respectively 4-fold- and 2-fold increased risk). However, for some other psychiatric disorders (e.g., eating disorders, developmental, and behavioral disorders), the overall number of cases was too few to perform the analysis or detect significant effects.

Organic disorders (e.g., demonstrable cerebral disease or brain injury) were the only psychiatric diagnosis that was significantly bidirectionally associated with BP, even after controlling for medications. According to a recent systematic review and meta-analysis [24] most studies provided evidence for neurological events preceding the development of BP [26, 30, 44, 45], but others reported high risk for some neurological disorders [46–48] in patients with a prior diagnosis of BP, during follow-up. Additional prospective cohort studies are required to clarify the temporal relation between BP and neurologic disorders. According to the current theory, the exposure of the BPAG1 neuronal antigen, following neurological damage or neuroinflammation, could trigger the production of autoantibodies that could cross-react with the epithelial BPAG1 isoform. Nevertheless, a similar pathophysiological mechanism could be hypothesized, given evidence of the involvement of immune activation in several of the psychiatric disorders associated with BP, such as schizophrenia [49], mood disorders [50], personality disorders [51], and substance use disorders [52].

Our work extends the literature by considering the role of both BP-related and psychiatric medications in increasing risk for the other class of illness. More than 50 medications have been identified to be associated with BP, with several explanatory theories proposed [53]. Drug-induced BP, a variant of idiopathic BP, can be clinically indistinguishable, and should be considered in older patients undergoing medication changes, as its course may be self-limiting when the medication is withdrawn. Although various diuretics are widely recognized to affect BP onset [29, 54–56], the association with psychiatric medications is less clear, in part due to methodological inconsistencies across studies. A register-based study examined the intake of psychiatric medications during the 2 years prior the diagnosis of BP and demonstrated that antipsychotics, antidepressants, and anxiolytics were significantly associated with BP [36]. These results agree with several previous findings [29, 33, 34, 57], but are in contrast with the study of Lloyd-Lavery et al. that stated that neither antipsychotics, antidepressants, nor anxiolytics were associated with BP [54]. Our findings show that psychiatric medications did not influence the risk of a subsequent BP diagnosis. Similarly, Bastuji-Garin and colleagues [29] considered medication intake as a potential confounding factor between mood disorders and BP, however, they restricted the multivariate analysis to psycholeptic medications, whereas we considered the whole class of psychiatric medications. It is possible that any effect on BP/ABD risk may not be equally distributed across psychoactive medications. Notwithstanding the majority of evidence supports the strong association between psychiatric medications and BP, we hypothesize that, even assuming the potential effect of medications, the association with psychiatric disorders is not only mediated by psychiatric medications.

By contrast, the medications prescribed for the treatment of BP/ABD accounted for the majority of risk of later psychiatric disorders. Systemic steroids are the first line treatment for BP/ABD [58] and general consensus exists regarding their ability to induce the onset or re-exacerbation of psychiatric symptoms and disorders [59, 60]. Psychiatric adverse effects typically consist of mild to moderate symptoms of anxiety, mood liability, and sleep disturbances that occur within the first week of treatment, are short-lasting, and reverse with the medication cessation [60]. However, serious conditions as delirium [61], psychosis [62, 63] and mood disorders [64–66] can occur, especially with high corticosteroid doses [59, 67].

Although the relation between corticosteroids and depression is well-documented, it is noteworthy that late-life depression can often result from multi-morbidity of somatic illnesses and polypharmacy [68, 69]. However, for cases that are plausibly related to corticosteroids, there is evidence for a dose-dependent effect and lower corticosteroid doses and cautious dosage reduction are recommended [70, 71].

To the best of our knowledge, this is the first study to explore psychiatric comorbidity in the whole class of ABD. The risk of a later BP or ABD was comparable for any psychiatric disorder. In contrast, the risk of developing a later psychiatric disorder after ABD was higher than BP.

Our study has strengths and limitations. First, the large sample size provided sufficient power for most comparisons of these relatively rare dermatologic diseases. Second, by using the Danish national registers, we likely captured the majority of incident cases during the observation period as the bullous disorders are sufficiently distressing to warrant detection, especially in a national healthcare system where treatment is essentially free and universally available. These strengths support the validity and generalizability of the results. However, some limitations have to be addressed. Firstly, the use of registry data did not allow to directly verify either the dermatologic or psychiatric diagnoses. Nonetheless, the Danish registries have undergone considerable validation and showed to be reliable for epidemiological research, with good internal validity and accuracy [39, 72, 73]. Furthermore, only data about BP and psychiatric disorders treated in secondary care (hospital inpatients and outpatients) were collected, with a plausible selection bias of more severe cases. We were underpowered to explore the association of less common psychiatric disorders with BP/ABD. Lastly, since we examined the combined effect of medications, we are not able to draw specific conclusions about the specific effect of individual medications.

In conclusion, the risk of developing BP after having a psychiatric disorder is elevated and is independent of psychiatric medications. Conversely, the association between prior BP and later psychiatric disorders seem to be primarily attributable to the use of medications for the treatment of BP. Clinically, an integrated approach attending to both dermatological and psychiatric symptoms is recommended. Dermatologists treating BP/ABD should remain vigilant and instruct patients under treatment about the possible development of both early and late psychiatric symptoms [59, 60], and refer them for psychiatric evaluation, when the medication cannot be reduced or discontinued [60, 74].

## Conclusions

Early detection, together with the modification of pharmacotherapeutic approaches to BP, could lower risk of developing subsequent psychiatric disorders. Future science should include even larger and more comprehensive prospective studies to confirm the temporal relation and the pathogenic mechanisms that underlie the association between psychiatric disorders and BP.

## Data Availability

Access to individual-level Denmark data is governed by Danish authorities. Each scientific project must be approved before initiation, and approval is granted to a specific Danish research institution. Researchers at Danish research institutions may obtain the relevant approvals and data access. International researchers may gain data access if governed by a Danish research institution having the needed approvals and data access.

## Abbreviations

BP: Bullous pemphigoid
ABD: All bullous disorders
BP Ag1: Bullous pemphigoid antigen 1
BP Ag2: Bullous pemphigoid antigen 2
OR: Odds ratio
ICD: International Statistical Classification of Diseases and Related Health Problems
ATC: Anatomical Therapeutic Chemical
HR: Hazard ratio
CI: Confidence interval
