# Correction to: Psychiatric comorbidity in individuals with bullous pemphigoid and all bullous disorders in the Danish national registers

**DOI:** 10.1186/s12888-020-02842-3

**Published:** 2020-09-04

**Authors:** Marianna Rania, Liselotte Vogdrup Petersen, Michael Eriksen Benros, Zhi Liu, Luis Diaz, Cynthia M. Bulik

**Affiliations:** 1 Department of Health Sciences, University Magna Graecia of Catanzar, Catanzaro, Italy; 2 Center for Clinical Research and Treatment of Eating Disorders, Mater Domini University Hospital, Catanzaro, Italy; 3 Department of Medical Epidemiology and Biostatistics, Karolinska Institutet, Stockholm, Sweden; 4 National Centre for Register-based Research, Aarhus BSS, Aarhus University, Aarhus, Denmark; 5 Centre for Integrated Register-based Research (CIRRAU), Aarhus University, Aarhus, Denmark; 6 Copenhagen Research Centre for Mental Health, Mental Health Centre Copenhagen, Copenhagen University Hospital, Copenhagen, Denmark; 7 Departments of Dermatology, Microbiology and Immunology, University of North Carolina at Chapel Hill, Chapel Hill, NC USA; 8 Department of Psychiatry, University of North Carolina at Chapel Hill, Chapel Hill, NC USA; 9 Department of Nutrition, University of North Carolina at Chapel Hill, Chapel Hill, NC USA

**Correction to: BMC Psychiatry 20, 411 (2020)**

**https://doi.org/10.1186/s12888-020-02810-x**

Following publication of the original article [1], the authors identified an error in the author name of Michael Eriksen Benros.

1. Incorrect name:

Michael Erikson Benros

2. Correct name:

Michael Eriksen Benros

The author group has been updated above and the original article [1] has been corrected.
